# Multi-Modal Dual-Task Measurement: A New Virtual Reality for Assessment

**DOI:** 10.3389/fpsyg.2020.635413

**Published:** 2021-01-22

**Authors:** Tom Burke, Brendan Rooney

**Affiliations:** School of Psychology, University College Dublin, Dublin, Ireland

**Keywords:** virtual reality, attention assessment, multimodal assessments, dual-task, psychometric development

## Introduction

This *Opinion Paper* considers the role of Virtual Reality (VR) as a viable platform for the clinical utility of dual-task assessments. VR has emerged as a promising tool for the diagnosis, treatment and cognitive improvement of neurological conditions, stroke, sport-related concussion, and dementia (Büttner et al., 2020; Sobral and Pestana, 2020). VR can be used in isolation for specific assessments of cognitive functions (Hørlyck et al., 2021), or can be combined with neuroimaging techniques and ancillary methods to offer insight into how target brain regions respond *in-vivo* to neurorehabilitation and/or cognitive stimulation (Ansado et al., 2020). The specific development of VR-based cognitive assessments and therapeutic interventions in neurological conditions is growing (Schultheis et al., 2002; Bell et al., 2020; Clay et al., 2020; De Luca et al., 2020; Diaz-Orueta et al., 2020; EbrahimiSani et al., 2020; Gamito et al., 2020; Vass et al., 2020). Within clinical practice, cognitive assessments rely on high levels of experimental control, and this is congruent with assessment paradigms in VR, while VR also potentially provides greater ecological validity than clinic-based assessments can provide (Parsons, 2015). The application of VR is becoming more commonplace in clinical trials and trial-based design (Clay et al., 2020; Escamilla et al., 2020; Hsieh et al., 2020), with some challenges reported in translating clinical trial outcomes to clinical practice (Brown et al., 2020).

Ideally, traditional neuropsychological assessments are developed using incremental levels of difficulty (Benson et al., 2010), to recruit additional cognitive processes as the test continues, or as serial stages of the test are administered to participants (Radua et al., 2014). For VR, this model of assessment has been mirrored with examples of VR-based tests including variable executive and functional levels of difficulty (Chang et al., 2020), which are also used as ecologically valid functional capacity assessments e.g., the Virtual Reality Functional Capacity Assessment Tool (Ruse et al., 2014). VR has shown clinically meaningful potential to provide opportunity for assessment and observation of motor function, gait analysis, motor imagery, and spatial aspects of cognition. While such assessment paradigms have been developed and used in neurodevelopmental conditions e.g., developmental co-ordination disorder (EbrahimiSani et al., 2020), there are exponential avenues for the development of clinical assessment for neuromuscular neurodegenerative diseases (Bekkers et al., 2020). Certainly, this is an emerging area within neurodegenerative movement disorders, such as Parkinson's Disease, or Multiple Sclerosis, where dual-task assessments are commonly used (Bekkers et al., 2020; Maggio et al., 2020; Saldana et al., 2020).

Dual-task assessments can be particularly useful from a clinical perspective, especially where a person who has difficulties with their gait, balance, and/or co-ordination, also presents with concurrent cognitive impairment or difficulties (Kalyani et al., 2019). Typically, people are able to perform motor and higher order cognitive tasks at the same time e.g., walking and holding a conversation. For some people with neurological conditions or brain injury, simultaneous performance of two such tasks often leads to performance deficits in one or both. This effect, known as dual-task interference, is thought to be evidence of deficits in selection and resourcing of cognitive function, at a given time. There are a number of proposed reasons for this; specific neurological conditions or head injury may result in domain-specific cognitive impairment due to the brain-behavior relationships associated with a specific neuroanatomical region (Bayot et al., 2018). This modular view of cognitive processing is challenged, however, by a more network-based approach to cognitive processing. For example, in isolation a person may be able to perform each individual task well e.g., walking without talking, though the capacity required to complete simultaneous tasks well, may be impaired. The extent to which the deficits are common to all tasks serves to indicate how general or specific the cognitive decline.

During the earlier stages of recovery from head or traumatic brain injury, performing a motor task may only be possible if the full computational resources of central cognitive capacity are available to direct and control movement. This would mean that the additional capacity available for any concurrent cognitive task is diminished, producing a dual task cognitive decrement. Similarly, for people with neurodegenerative movement disorders like Parkinson's Disease, dual-task interference can manifest in the everyday difficulties observed with executing both cognitive and motor tasks simultaneously (Raffegeau et al., 2019).

## Literature Gap

In current dual-task paradigms, there appears to be a high level of sophistication with motor assessment and outcomes, with non-specific cognitive measures being used to supplement the motor assessment (Veldkamp et al., 2019). Typically, to facilitate this assessment, headphones are used to administer the test stimuli, which allows for a motor assessment e.g., walking, concurrent with an attention task e.g., auditory digit-span. To that end, attention assessments to date during dual-task assessments have been restricted to auditory input, with little to no assessments of spatial span. Pragmatically, this is intuitive as the participant is required to use visual scanning to navigate their environment for safety while completing the task. Notwithstanding, the development of a multi-modal dual-task assessment is needed to overcome this current clinic-based limitation, allowing for a valid assessment of spatial span during a movement task. This in turn, could provide more sophisticated technologically supported assessments of auditory attention which may be more informative for clinical trials.

## Discussion

Emerging technology, such as virtual reality, can provide cognitive assessments with number of important benefits. VR assessments are flexible so they can be tailored with precise adjustment to meet the exact needs for a specific person, yet standardized for benchmarking performance. In this way they can powerfully inform individual rehabilitation support needs and strategies. In addition, their multi-modal nature offers a broad range of neuropsychological domains, within which assessment can take place and offers a way to mimic real world environments so as to embed the assessments in contextualized and meaningful activities. Through the use of a VR assessment-based model, one could tailor a motor task to the person and their current capacity, then concurrently administer a graded measure of auditory or visual attention, tailored to the person. Through the use of VR, the interaction between motor control and cognitive demand can be assessed with high levels of experimental control for the clinician-scientist, without compromising the ecologically-validating assessment experiences that support rehabilitating patients. On the other hand, an assessment paradigm such as this may also provide useful clinical markers for neurodegenerative conditions where dual-tasks are routinely used, such as Parkinson's Disease. Thus, this *Opinion Paper* supports the consideration of not only dual-tasks to be considered within VR, but for multi-modal and cross-modal cognitive-motor assessment paradigms to be considered. This has implications for clinical assessment and intervention, as the degree of interference between motor and cognitive tasks may be a potential indicator of the functional state of the motor system, cognitive function, and indeed the integration of both, during rehabilitation.

The development of a multimodal dual-task VR assessment which is underpinned by cognitive models of attention and working memory will be a great strength to cognitive assessment through the use of VR going forward with aging populations and the development of technology. Such an assessment paradigm would allow the facilitation of attention and working memory tasks, through multi-modal delivery e.g., auditory digit span and visual spatial span. To date, few studies, have specifically investigated the fractionation of attention and working memory processes stratified by input modality (Ettenhofer et al., 2016). Yet such an approach is of great importance when considering neurological conditions or brain injury. For example, previous research has demonstrated relatively independent patterns of performance on the standard Spatial Span and digit span tasks (Wilde and Strauss, 2002; Flaks et al., 2014). This lends support to the vast work demonstrating the relative separation of multimodal processing in theories of attention and working memory (Baddeley and Hitch, 1994; Baddeley, 1996, 2000, 2001, 2003, 2012; Bruyer and Scailquin, 1998; Repovs and Baddeley, 2006; Baddeley et al., 2011, 2019). While on a cursory methodological level, traditional versions of these tasks may show analogous properties, there is doubt about whether they show similar operating characteristics and measure the same cognitive process.

To elaborate, from a cognitive model perspective, where a task requires target stimuli of increasing length to be reproduced in the order they were presented, the task evaluates attention through modality-specific systems i.e., the phonological loop and the visuo-spatial sketchpad for verbal and visual or spatial data, respectively. Whereas if a task requires stimuli to be manipulated (e.g., reversed prior to reproduction), then this task primarily implicates executive function, as part of the same modality-specific systems, and is consequently considered a working memory task. For this reason comprehensive assessments require specific tasks matched on equivalency. Here, we propose that VR can provide a novel avenue to consider a multimodal cognitive assessment. Importantly, this can be combined with a motor task to develop a VR-specific dual-task assessment.

Potential dissociation between impaired attention (verbal and/or visuospatial) and short-term memory may be of most importance when assessing impairment in early stages of a disease, prior to impairment of the wider executive system. A VR-based multi-modal dual-task could assess whether patients' patterns of scoring on dual-tasks, are equivalent on measures of attention and working memory, regardless of the modality used i.e., auditory (digit-span) or visual (spatial span). From a clinical perspective, such a measure allows for a person to complete an assessment as they present to a clinic at one timepoint, and then reliably repeat the assessment over time. Importantly, the modality could be changed in light of any progressive motor impairment e.g., using a spatial task rather than one relying on verbally responding to tasks. The conceptualization of this task has not only clinical importance for dual-task development, but also for the development of cross-modal tasks. It is expected that tasks such as this will provide valid, reliable, and clinically meaningful results for conditions where dual-task paradigms are currently being used, in a more traditional modality. The technologically-supported VR dual-task, with multimodal components, can also be designed to collect a range of performance and outcome metrics such as rapid response latency, accuracy, error monitoring, and recordings of data relating to the relationship between motor and cognitive outcomes. These data may uncover more reliable indices of functioning, diagnosis and prognosis. Notwithstanding, there are a number of potential limitations for consideration which may have a secondary influence of participants' performance, which are related to the use of VR, over and above a clinical syndrome. Potential negative affects need due consideration, and these may include health and safety risks e.g., visually-induced motion sickness (cybersickness; Arcioni et al., 2019), challenges to performance e.g., discomfort with head mounted displays and equipment (Zhdanov et al., 2019), or social implications e.g., the acceptability of VR and VR-based assessments (Stanney et al., 1998). Consequently, clinician scientists need to ensure gold standard assessments and cross-validations occur with the introduction of new VR-based assessments, in order to provide reliable and valid metrics, as well as VR-alternatives should a person find it challenging or comfortable to engage with VR.

This article forms part of a special issue on the use of neuropsychological and cognitive assessment in neurodegenerative diseases, through the use of VR. From a clinical perspective, attention and working memory tasks have an important role to play in the cognitive assessment of neurodegenerative disorders, especially where motor function is a core feature e.g., Huntington's disease. The combination of verbal and visuospatial span tasks are of great importance when examining attention and working memory, yet traditionally, a single test modality is often chosen. Clinically, measures of attention, executive function, and short-term memory have important prognostic value in neurodegenerative syndromes, and a multimodal dual-task such as this will have particular utility in motor conditions involving cortico-striatal-thalamo-cortical pathways e.g., Parkinson's Disease (Peters et al., 2016). To integrate a VR-based assessment paradigm into clinical practice, a novel measure is required to have cross-modal interchangeability to support dynamic patient presentations in neurodegenerative movement disorders. From a usability perspective, it is also required to be portable, and cost-effective. There is vast scope for dual-task assessments through the use of VR in neurodegenerative and neurological conditions, and this *Opinion Paper* highlights some of the cognitive and neuropsychological considerations to be made, as well as potential avenues for outcomes specific to assessing auditory and visual attention.

## Author Contributions

Both authors contributed equally to this Opinion Paper.

## Conflict of Interest

The authors declare that the research was conducted in the absence of any commercial or financial relationships that could be construed as a potential conflict of interest.
